# Comprehensive Analysis of Prognostic Alternative Splicing Signature Reveals Recurrence Predictor for Papillary Thyroid Cancer

**DOI:** 10.3389/fonc.2021.705929

**Published:** 2021-10-13

**Authors:** Mian Liu, Rooh Afza Khushbu, Pei Chen, Hui-Yu Hu, Neng Tang, Deng-jie Ou-yang, Bo Wei, Ya-xin Zhao, Peng Huang, Shi Chang

**Affiliations:** ^1^ Department of General Surgery, Xiangya Hospital Central South University, Changsha, China; ^2^ Clinical Research Center for Thyroid Disease in Hunan Province, Changsha, China; ^3^ National Clinical Research Center for Geriatric Disorders, Xiangya Hospital, Changsha, China

**Keywords:** alterative splicing, papillary thyroid cancer, recurrence-free survival, prognosis, splicing factor

## Abstract

**Background:**

Alternative splicing (AS) plays a key role in the diversity of proteins and is closely associated with tumorigenicity. The aim of this study was to systemically analyze RNA alternative splicing (AS) and identify its prognostic value for papillary thyroid cancer (PTC).

**Methods:**

AS percent-splice-in (PSI) data of 430 patients with PTC were downloaded from the TCGA SpliceSeq database. We successfully identified recurrence-free survival (RFS)-associated AS events through univariate Cox regression, LASSO regression and multivariate regression and then constructed different types of prognostic prediction models. Gene function enrichment analysis revealed the relevant signaling pathways involved in RFS-related AS events. Simultaneously, a regulatory network diagram of AS and splicing factors (SFs) was established.

**Results:**

We identified 1397 RFS-related AS events which could be used as the potential prognostic biomarkers for PTC. Based on these RFS-related AS events, we constructed a ten-AS event prognostic prediction signature that could distinguish high-and low-risk patients and was highly capable of predicting PTC patient prognosis. ROC curve analysis revealed the excellent predictive ability of the ten-AS events model, with an area under the curve (AUC) value of 0.889; the highest prediction intensity for one-year RFS was 0.923, indicating that the model could be used as a prognostic biomarker for PTC. In addition, the nomogram constructed by the risk score of the ten-AS model also showed high predictive efficiency for the prognosis of PTC patients. Finally, the constructed SF-AS network diagram revealed the regulatory role of SFs in PTC.

**Conclusion:**

Through the limited analysis, AS events could be regarded as reliable prognostic biomarkers for PTC. The splicing correlation network also provided new insight into the potential molecular mechanisms of PTC.

## Introduction

Thyroid cancer is the most rapidly increasing malignancy worldwide in both men and women (1). Papillary thyroid cancer (PTC), the most common thyroid carcinoma type, comprise 80% of all cases (1). PTC has several subtypes, including classical papillary cancers, less aggressive variants, such as follicular, oxyphilic, and cribriform-morular variants, and more aggressive variants, such as diffuse-sclerosing, tall-cell, columnar-cell and solid variants (2). In general, PTC has an excellent prognosis, with a 5-year survival rate over 97%, and PTC tumors measuring less than 1 cm have a 10-year disease-specific survival rate over 99% (3). Although PTC is associated with low mortality, the incidence of disease recurrence or metastasis is 20-30%, and is even higher in patients with the more aggressive variants (4, 5). It is important to assess the PTC recurrence risk accurately for ensuring patients to receive the most appropriate treatment strategy. Over the past few decades, great efforts have been made in exploring prognostic biomarkers for PTC, in particular gene markers, such as mutations in the *BRAF, RAS, PIK3CA, P53, PTEN, P53* and *ALK* genes (6, 7). Although these studies showed promising results, they only focused on the factors driven by mutation and the level of transcription while ignoring the diversity of RNA isoform resulting from posttranscriptional modifications. Currently, there is no consensus on assessing the prognosis of PTC patients.

Alternative splicing (AS) is a significant molecular posttranscriptional modification mechanism that converts mRNA into different RNA transcripts which are then translated into different protein products, thus greatly increasing the diversity of protein species (8, 9). Recent studies have shown that more than 90% of human genes have AS modifications and these modifications play an important role in biological processes (10, 11). The dysregulation of AS is involved in a variety of physiological and pathological processes, including tumorigenesis. Since tumor cells tend to generate sub-isoform changes, which lead to the functional loss of tumor suppressor genes and the activation of oncogenic genes (12). These multiple AS events are conducive to tumor cell proliferation, invasion and metastasis, drug resistance and immune escape (13). For example, exon 13 skipping in CD46 and the exon 13-containing CD46 isoform play opposite roles in bladder cancer development, and exon 13 skipping remarkably accelerated DNA synthesis, cancer cell proliferation, migration and invasion (14). KLF6-SV1, an oncogenic alternatively-spliced isoform of KLF6 produced by alternative 5′ splice sites, is often highly expressed in various human malignancies including non-small cell lung cancer and hepatocellular carcinoma (15–17). *BRCA1/2* germline mutations are most commonly seen in breast and ovarian cancer patients who benefit from treatment with PARP inhibitors (PARPis) or platinum compounds, but BRCA1-Δ11q splice variants lacking the majority of exon 11 contribute to therapeutic resistance (18, 19). We found that prognostic models constructed from AS events data had good efficiency for evaluating the survival time of adrenocortical carcinoma, cervical cancer and prostate cancer patients (20–22).

In addition, some studies have shown that AS events could be intricately regulated by key splicing factors (SFs) (23). The abnormal expression of SFs cause subversive alteration in tumor-specific AS events, which affects the initiation and progression of carcinoma (24). In recent years, the development of genome-wide sequencing technology has provided new opportunities to explore and identify tumor-specific molecules and prognostic markers (25, 26). A comprehensive analysis of AS events and underlying SF-AS regulatory networks can provide new insight into the molecular mechanism of PTC and prognosis-related biomarkers for PTC patients. Preliminary studies in AS have provided evidence of prognosis value, while the function and mechanism of AS in PTC remains unknown.

In our study, we revealed a large number of RFS-related AS events in PTC through a systemic analysis of the AS events of all genes in the PTC cohort from the TCGA SpliceSeq dataset. We constructed a prognostic prediction model based on the identification of RFS-associated AS events, and presented the clinicopathological characteristics and a nomogram of AS prognostic predictors, which could predict the recurrence-free survival rate of PTC patients. Finally, development of an SF-AS relationship network diagram showed the potential regulatory mechanisms involved in PTC recurrence and patient prognosis.

## Materials And Methods

**Figure 1:**
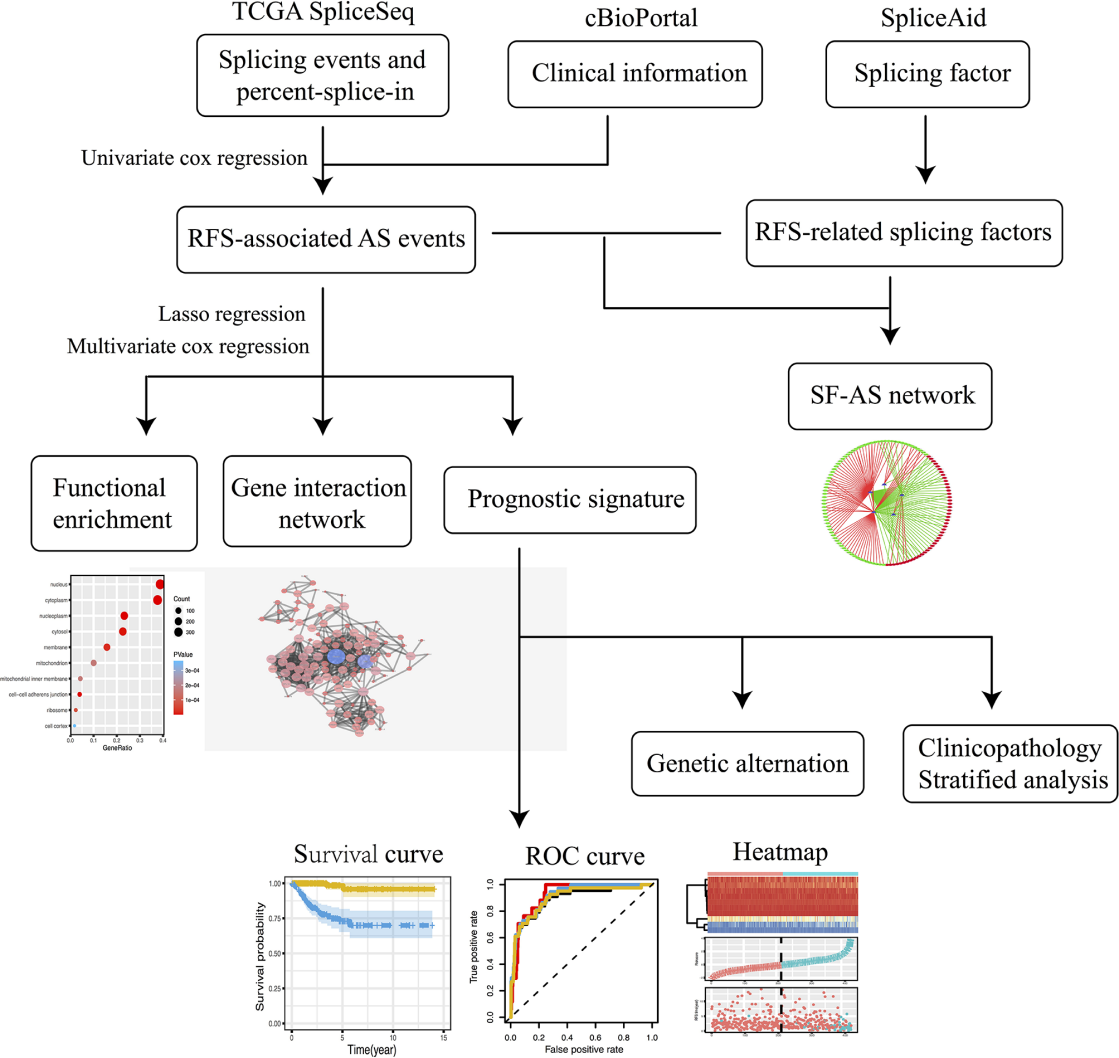
The flowchart of the study.

The flowchart of the study is shown in **Figure 1**.

### Acquisition of AS Data

The percent splice-in (PSI) data of AS events in PTC were downloaded from the TCGA SpliceSeq (https://bioinformatics.mdanderson.org/TCGASpliceSeq/) database, which provides the overview of AS events across 33 types of tumors based on TCGA RNA-seq data. AS events for 7 types have been identified so far, namely Alternate Acceptor site (AA), Alternate Donor site (AD), Alternate Promoter (AP), Alternate Terminator (AT), Exon Skip (ES), Mutually Exclusive Exons (ME) and Retained Intron (RI) (27). The annotation of the AS event consists of the parent gene symbol, the unique ID number and the splicing type. PSI values are used to quantify AS events, and its range is from zero to one. In order to acquire a credible AS events data set, we set a strict screening condition that the proportion of samples contain PSI values over 75%. We filtered out the data with AS events missing rate over than 20%, then replaced the missing value with median values.

### Analysis of RFS-Related AS Events, Function and Pathway Enrichment Analysis and Gene Interaction Network

The clinical information of the PTC cohort was obtained from the cBioPortal (http://www.cbioportal.org) database, including recurrence-free survival status and time. Patients were divided into high and low PSI subgroups based on the median value of PSI, and then univariate Cox regression analysis was used to explore RFS-associated seven types AS events respectively, p values less than 0.05 considered as statistically significant. UpSetR mapping was used to analyze the interaction between the RFS-related AS events for each splicing type and corresponding parent genes. Target genes network were constructed *via* the Search Tool for the Retrieval of Interacting Genes (STRING, https://string-db.org) and Cytoscape (version 3.7.1). Database for Annotation, Visualization and Integrated Discovery (DAVID) online functional annotation tool (https://david.ncifcrf.gov/tools.jsp) was used to complete genetic function and pathway enrichment analysis, and use RStudio drawing.

### Construction of the AS Model for Predicting Recurrence of PTC Patients

First, LASSO regression analysis was performed on the RFS-associated AS events obtained of 7 types by univariate Cox regression analysis. In order to avoid overfitting of the model, multivariate Cox regression analysis was used to further screen the candidate AS events and identify independent prognostic predictors. We calculated the risk score for each patient based on each predictor and the calculation formula is as follows: Risk score = PSI_AS event1_ × coefficient _AS event1_+ PSI_AS event2_ × coefficient _AS event2_+· · · + PSI_AS eventn_ × coefficient _AS eventn._ According to the median risk score, PTC patients were divided into high and low risk subgroups, and Kaplan-Meier analysis was used to evaluate the accuracy of each prognostic prediction signature. In addition, the receiver operating characteristic (ROC) curve by the survival ROC package was used to calculate the corresponding area under the curve (AUC) value. Furthermore, the cBioPortal online database was used to analyze mutations and expression changes in corresponding parental genes.

### The Verification of Prognostic Value of AS Predictor

The modeling dataset was random divided into two validation datasets (50 percent vs 50 percent, n=215), Kaplan-Meier survival curve and ROC curve were used to evaluate the performance of the model. Besides, we also performed a pan-cancer* *survival analysis based on data from TCGA.

To further analyze the independent risk factor associated with recurrence of PTC, AS prognostic predictor signature along with all clinicopathological variable mentioned above were performed by univariate Cox regression analysis. The candidate variables were subjected to multivariate regression analysis to screen out independent prognostic predictors.

In addition, we analyzed the clinicopathological characteristics of the high- and low-risk subgroups. Judge and verify the prognostic performance of the final AS prediction model in the stratified survival analysis, such as age, sex, histologic subtype, tumor grade, lymph node grade, and pathological stage.

### Analysis of RFS-Related SFs and Construction of SF-AS Relationship Network

Splicing factors (SFs) were obtained from SpliceAid 2 (www.introni.it/spliceaid.html) database. The normalized mRNA expression data of the SFs were obtained from UCSC Xena (https://xena.ucsc.edu) database. The Protein expression level of SFs was obtained from The Human Protein Atlas (https://www.proteinatlas.org/) database. Univariate Cox regression analysis was used to screen out RFS-related SFs. Spearman correlation analysis was used to detect the relationship between RFS-related AS events and SFs, P value less than 0.05 and the correlation coefficient greater than 0.4 as cutoff value. Finally, Cytoscape is used to construct a potential SF-AS relationship network diagram.

## Results

### A Complete Overview of AS Events in the TCGA PTC Cohort

Through integrating all AS events of PTC patients from the TCGA SpliceSeq database, we discovered 37833 AS events involving 18231 genes, including 10219 ESs in 3904 genes, 9127 APs in 3653 genes, 8597 ATs in 3753 genes, 3683 AAs in 2592 genes, 3190 ADs in 2240 genes, 2787 RIs in 1865 genes, and 232 MEs in 224 genes (see **Supplementary Figure 1A**). The figures showed that one gene can produce multiple types of AS events in PTC patients. Among these 7 types of AS events, the most frequent splicing type was ES, while the least type was ME (see **Supplemental Figure 1A**).

### Detection of RFS-Related AS Events and Analysis of Function and Pathway Enrichment

**Figure 2:**
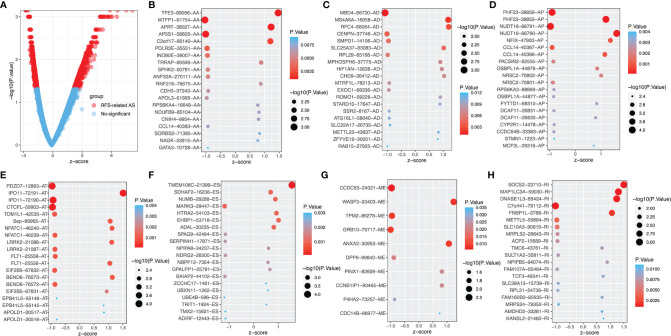
The top 20 RFS-associated AS events. **(A)** Volcano map of AS event, red dots represent RFS- related AS. The top 20 AS events related to recurrence outcomes in different splice types in PTC, including **(B)** AA, alternate acceptor site. **(C)** AD, alternate donor site. **(D)** AP, alternate promoter. **(E)** AT, alternate terminator. **(F)** ES, exon skip. **(G)** ME, mutually exclusive exons. **(H)** RI, retained intron.

**Figure 3:**
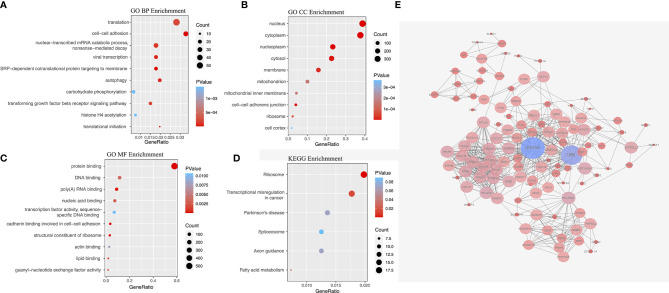
The gene interaction network of RFS-associated AS events, functional and pathway analysis. **(A)** GO biological processes (BP) enrichment. **(B)** GO cellular component (CC) enrichment. **(C)** GO molecular function (MF) enrichment. **(D)** KEGG pathway enrichment analysis. **(E)** Parent genes interaction network.

The survival and clinical information for PTC was obtained from the cBioPortal database (**Supplementary Table 1**). There was a total of 430 PTC patients with available recurrence-free survival time data and complete clinical information in our analysis. In PTC cohort, univariate Cox analysis of all AS events revealed that 1396 AS events were significantly related to the RFS (P<0.05, **Supplementary Tables 2**). In order to better visualize the intersection of different types of AS events and corresponding parent genes, an UpSet plot was constructed, as shown in **Supplementary Figure 1B**. Interestingly, we found that one gene can produce 3 different types of AS events in this study. The different types of prognoses associated AS events, except ME, in the top 20 genes are clearly exhibited in **Figure 2**. Next, we performed functional and pathway enrichment of 989 parent genes of RFS-associated AS events. The results showed that a total of 130 GO terms and 3 KEGG terms were significantly involved in prognosis (p<0.05), and **Figures 3A**–**D** showed the top 10 GO functional enrichment and KEGG pathways. To further explore the biological association between the corresponding paternal genes in PTC, we used STRING and Cytoscape to create a gene interaction network. **Figure 3E** shows a network diagram of the parental genes. The larger the node, the greater the degree of association with other genes, and the top 3 genes identified were UBA52, UBB and RPL31, they may be closely related to the occurrence and progression of PTC.

### Establishment of AS Recurrence Prediction Model for PTC Patients

**Figure 4:**
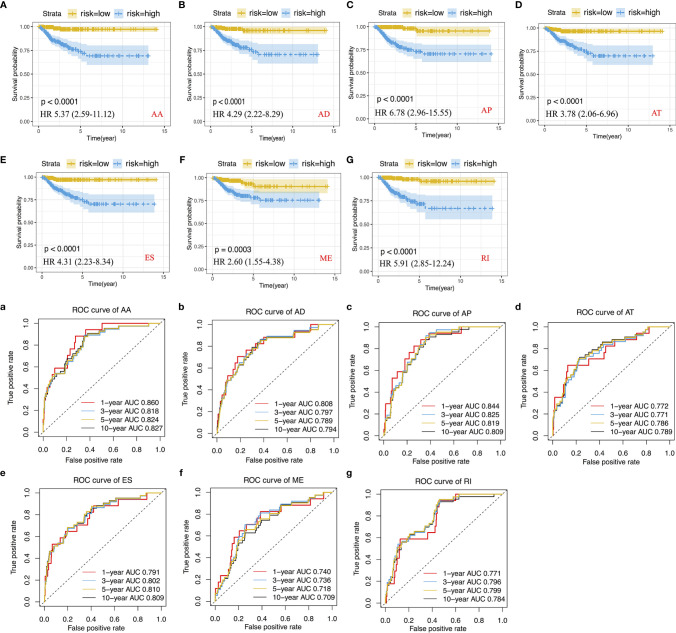
Construction of Kaplan-Meier survival curve and ROC curve and calculation of AUC values ​​for recurrence prognostic predictors. **(A–G)** Kaplan-Meier survival curve for AA, AD, AP, AT, ES, ME and RI prediction models. **(a–g)** ROC curve for AA, AD, AP, AT, ES, ME and RI prediction models.

**Figure 5:**
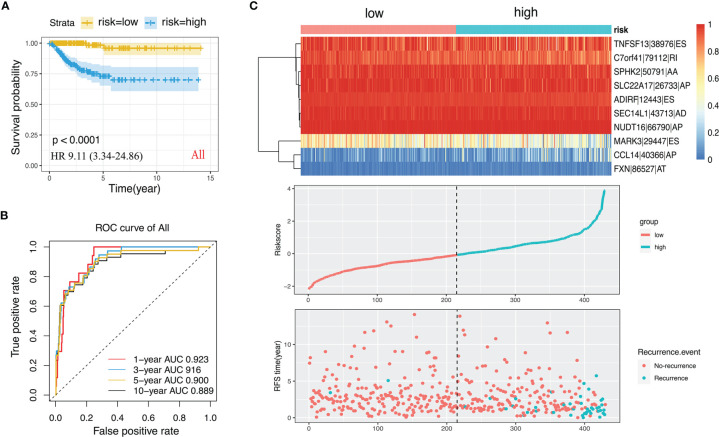
The ten-AS events signature as the best prognostic predictive model in PTC cohort. **(A)** Kaplan-Meier curve of the ten-AS prognostic predictor. **(B)** ROC curve of the ten-AS prognostic predictor. **(C)** The upper part showed the heatmap of PSI score of AS events involved in the prognostic predictor. The middle part was risk score of each individual. The bottom was the recurrence status and RFS time of each PTC patients.

We performed LASSO regression analysis for the significant RFS-associated AS events in each AS type (**Supplementary Figure 2A**–**G**). In order to avoid model overfitting, the above results of each AS type were further analyzed by multivariate Cox regression analysis to screen out the most suitable predictor for AS recurrence models. Seven types of AS models (AA, AT, ME, RI, AD, AP and ES) were constructed, and the formula corresponding to each model was shown in **Table 1**. Based on the formula, we calculated the risk score of each patient and divided into high and low risk groups. The Kaplan-Meier survival analysis showed that the recurrence model of each AS type had good predictive power to distinguish between good and poor survival results (**Figures 4A**–**G**). To further evaluate and compare the efficiency of the model, ROC curves were used to calculate the AUC value predicting the 1-year, 3-year, 5-year and 10-year recurrence-free survival rate (**Figures 4a**–**g**). The AUC values ​​of the seven types of models at different times did not exceed 0.04. The largest AUC value of the ROC for the 1-year, 5-year and 10-year RFS rate was obtained with the AA prognostic predictor (0.860, 0.824 and 0.827 respectively), and the largest AUC value for the 3-year survival rate was obtained with the AP model (0.825). Importantly, a ten-AS event predictor was obtained by the overall analysis of prognostic-related AS events using LASSO regression and multivariate Cox regression analysis (**Supplementary Figure 2H**). The calculation used for the risk score is shown in **Tables 1** and **2**, the high-risk group showed a worse significant survival outcome than low-risk (**Figure 5A**). The 1-year, 3-year, 5-year and 10-year AUC values of the ROC curve for the combined model were calculated as 0.923, 0.916, 0.900 and 0.889 respectively. These values were higher than those obtained by the seven separate models individually, suggesting that the mixed AS model had the highest-level performance among all prognostic models (**Figure 5B**). The distribution of patient survival status and survival time, risk score for the prognostic predictors and the PSI of the ten AS events for final recurrence model, as illustrated in **Figure 5C**, the results showed that the shorter the patient’s survival time and the more recurrent cases, the higher the risk score of the model was significantly higher (P <0.05, **Figure 5C**).

**Table 1:** Formula of each prognostic signature for PTC.

Type	Formula
**AA**	DGKZ|15545|AA× (-2.68) + ZC3H14|28713|AA× (1.44) + LRRC28|32640|AA× (-1.20) + INO80E|36008|AA× (-4.92) + APRT|38027|AA× (5.86) + INO80C|45175|AA× (0.09) + SPHK2|50791|AA× (-4.97) + NR4A2|55620|AA× (1.23) + AP5S1|58605|AA× (-2.60) + CHCHD10|61315|AA × (-1.08) + MTFP1|61754|AA × (-1.17)
**AD**	STARD10|17647|AD× (0.45) + N4BP2L2|25597|AD × (-4.02) + SPINT1|30056|AD × (-0.48) + CHD9|36412|AD × (0.68) + IFI35|41177|AD× (-0.05) + SEC14L1|43713|AD × (-2.90) + MBD4|66720|AD × (-3.60)
**AP**	SLC22A17|26733|AP× (-2.64) + DCAF11|26830|AP × (0.22) + CCL14|40366|AP × (1.51) + NUDT16|66790|AP × (-22.52)
**AT**	FAM72A|9578|AT × (0.41) + ABCC4|26108|AT × (2.78) + SUPT16H|26571|AT × (5.75) + TOM1L1|42535|AT × (-4.95) + GDPD1|42768|AT × (7.27) + Mar|73177|AT × (-0.44) +COBL|79728|AT × (2.49) + FXN|86527|AT × (8.97) + KIF4A|89372|AT × (-0.06)
**ES**	PTER|10876|ES × (0.40) + ADIRF|12443|ES × (-24.30) + CS|22419|ES × (0.84) + C14orf159|28857|ES × (4.56) + NDUFB1|28987|ES × (3.98) + MARK3|29447|ES × (-1.31) + COX5A|31814|ES × (-3.68) + NPIPA8|34237|ES × (1.35)
**ME**	CCDC53|24021|ME × (-1.02) + WASF3|93403|ME × (-1.12)
**RI**	SOCS2|23710|RI× (0.35) + USHBP1|48248|RI × (0.89) + DNASE1L3|65424|RI × (0.07) + MTURN|79112|RI × (-3.98) + FAM160B2|82935|RI × (1.44)
**All**	SPHK2|50791|AA × (-2.33) + SEC14L1|43713|AD × (-0.42) + SLC22A17|26733|AP × (-0.21) + CCL14|40366|AP × (1.02) + NUDT16|66790|AP × (15.52) + FXN|86527|AT × (6.72) + ADIRF|12443|ES × (-23.02) + MARK3|29447|ES × (-0.69) + TNFSF13|38976|ES × (-0.29) + MTURN|79112|RI × (-3.57)

**Table 2:** Prognostic predictors for PTC.

Gene	AS id	type	Exons	HR	Lower95	Upper95	P-value	Index
**SPHK2**	50791	AA	3.3:3.4	0.51	0.32	0.82	0.005	-2.33
**SEC14L1**	43713	AD	4.2	0.57	0.35	0.9	0.017	-0.42
**SLC22A17**	26733	AP	1	0.54	0.34	0.85	0.008	-0.21
**CCL14**	40366	AP	1	2.41	1.45	4	0.001	1.02
**NUDT16**	66790	AP	2	2.86	1.66	4.92	0.0001	15.52
**FXN**	86527	AT	7	1.57	1.01	2.44	0.047	6.72
**ADIRF**	12443	ES	2	0.48	0.3	0.79	0.01	-23.02
**MARK3**	29447	ES	17	0.45	0.27	0.72	0.001	-0.69
**TNFSF13**	38976	ES	2.2:3	0.52	0.32	0.82	0.005	-0.29
**MTURN**	79112	RI	4.2	0.46	0.28	0.73	0.001	-3.57

**Figure 6:**
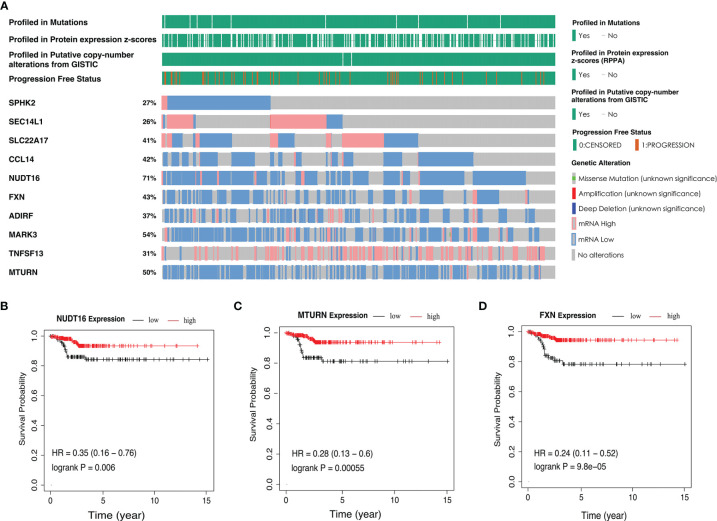
The parent genetic alteration in PTC cases. **(A)** In the PTC cohort, waterfall plot of parent genes variation and expression from the ten-AS models. **(B–D)** Kaplan-Meier survival curves of NUDT16, MTURN and FXN gene expression.

In addition, parental genetic alteration of the ten-AS event model is shown in **Figure 6A**. The mutation of these ten genes rarely appeared in PTC patients from the TCGA dataset, but the mRNA expression level of most of the genes were altered; for example, *NUD16* expression was decreased in 71% of PTC samples compared to normal tissue (**Figure 6A**). We detected the relationship between the expression level of parental genes and the RFS rate of PTC patients. There was a statistically significant relationship between PTC patient’s prognosis and the expression of *SPHK2, SLC22A17, NUDT16, FXN, ADIRF, MARK3* and *MTURN*. The representative survival curves showed that *NUDT16*, *MTURN* and *FXN* had the most changes, and high expression was a favorable prognostic factor (**Figures 6B**–**D**, **Supplementary Figure 3**). The changes in mRNA may be caused by AS events, but AS events are not limited to changes in mRNA levels as they are also involved in the specific functions of protein regions.

### The Efficiency of AS Prognostic Predictor in Stratified Clinicopathologic Subgroups in PTC Patients

According to the ten-AS prognostic model, PTC patients were divided into two risk levels (high or low). The clinicopathologic characteristics of the two groups as shown in **Table 3**, PTC patients with high risk tended to be over the age of 55 and had tumors with a higher grade (p<0.05). Moreover, we analyzed the predictive performance of ten-AS prognostic model in stratified PTC patients (**Table 3**). The prognostic model identified high-risk patients with worse RFS rates in each subgroup except T1 and Stage II, which may result from the small number of endpoint events in these two subgroups (**Table 3**). In order to further explore the potential factors related to recurrence in PTC patients, the clinicopathologic variables along with the risk score of the ten-AS prognostic predictor were subjected to univariate Cox regression analysis. Age <55, female sex, histologic type-classical PTC, pathologic T1, pathologic N1 and pathologic stage I were set as references. The results showed that age, tall cell type, T3, T4, N1b, stage III, stage IV and risk score were significantly related to PTC recurrence (**Table 4**). Furthermore, the meaningful factors were analyzed by multivariate Cox regression analysis, and the results uncovered that the ten-AS prognostic model was the only independent recurrence prognostic factor (**Table 4**). The risk score of the ten-AS model, the independent predictive factor, was used to establish nomogram (**Supplementary Figure 4**). Our results suggested that the ten-AS prognostic predictor had a better efficiency in predicting PTC recurrence than clinicopathological characteristics, and predictive value in stratified subgroups well.

**Table 3:** Clinicopathology feature of the final AS signature and prognostic analysis in stratified PTC cohorts.

	Low-risk (cases)	High-risk (cases)	P.value	Survival (P.value)
**Age**			**0.0054**	
**<55**	159	132		<0.0001
**≥55**	56	83		<0.0001
**Gender**			0.126	
**Female**	151	165		<0.0001
**Man**	64	50		<0.0001
**Subtype**			0.538	
**Follicular**	44	36		<0.0001
**Classical**	155	163		0.013
**Tall Cell**	19	16		0.0021
**Pathologic T**			**0.0087**	
**T1**	80	48		0.098
**T2**	62	76		0.0011
**T3**	64	82		<0.0001
**T4**	9	9		<0.0001
**Pathologic N**			0.613	
**N0**	112	109		<0.0001
**N1**	27	28		0.03
**N1a**	46	39		0.0056
**N1b**	30	39		0.0035
**Stage**			0.168	
**Stage I**	132	111		0.0001
**Stage II**	16	25		0.11
S**tage III**	46	51		0.0004
**Stage IV**	21	28		0.001

Variables with statistical significance were shown in bold.

**Table 4:** Univariate and multivariate Cox regression analysis for clinicopathology variables.

	Univariate analysis			Multivariate analysis		
	HR	(95% CI)	P-value	HR	(95% CI)	P-value
**Age**						
<55	Ref			Ref		
≥55	1.75	1.14-2.67	**0.01**	0.68	0.33-1.4	0.296
**Gender**						
**Female**	Ref			/		
**Man**	1.42	0.75-2.68	0.285	/	/	/
**Subtype**						
**Follicular**	Ref			Ref		
**Classical**	1.4	0.54-3.59	0.49	1.22	0.39-3.82	0.731
**Tall Cell**	3.45	1.09-10.89	**0.035**	2.1	0.47-9.34	0.329
**Pathologic T**						
**T1**	Ref			Ref		
**T2**	2.7	0.87-8.39	0.085	1.26	0.37-4.28	0.71
**T3**	5.04	1.74-14.59	**0.003**	1.77	0.53-5.91	0.354
**T4**	6.28	1.56-25.18	**0.01**	1.31	0.22-7.7	0.762
**Pathologic N**						
**N0**	Ref			Ref		
**N1**	2.05	0.89-4.69	0.091	2.14	0.75-6.06	0.153
**N1a**	1.51	0.66-3.45	0.329	0.99	0.38-2.62	0.99
**N1b**	2.65	1.19-5.92	**0.017**	2.82	0.96-8.29	0.059
**Stage**						
**Stage I**	Ref			Ref		
**Stage II**	1.05	0.31-3.61	0.935	1.02	0.2-5.15	0.979
**Stage III**	2.53	1.25-5.12	**0.01**	2.03	0.68-6.11	0.207
**Stage IV**	3.52	1.55-7.98	**0.003**	0.99	0.29-3.35	0.986
**Risk score**	16.06	9.58-26.9	**<0.0001**	20	10.23-39.11	**<0.0001**

Variables with statistical significance were shown in bold.

### The Verification of Prognostic Value of the Ten-AS Signature

Internal validation in two datasets shown good performance of the ten-AS signature, PTC patients with high glycolysis scores exhibited worse prognosis (see **Supplementary Figure 5**). Besides, we also evaluated the prognostic values of the ten-AS prognostic model in various cancers. In our results, the ten-AS prognostic model also applied to prostate adenocarcinoma (PRAD) and lung adenocarcinoma (LUAD), PRAD or LUAD patients with high-risk scores exhibited worse prognosis (see **Supplementary Figure 6**).

### Detection of RFS-Associated SFs and Construction of SF-AS Relationship Network

**Figure 7:**
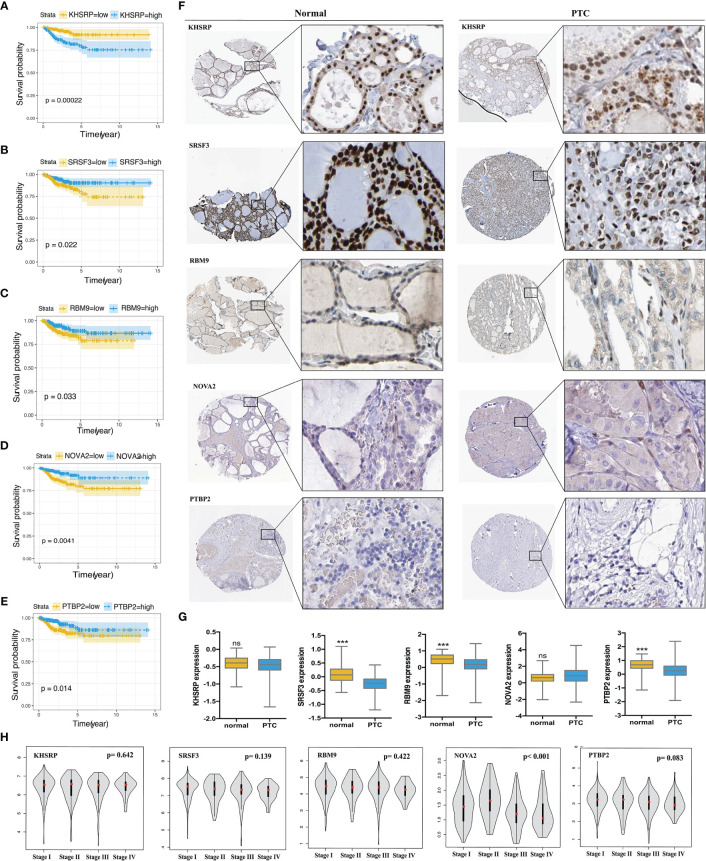
Validation the prognostic correlation of the targeting SFs and the expression in PTC tissues. Kaplan-Meier curves of **(A)** KHSRP **(B)** SRSF3 **(C)** RBM9 **(D)** NOVA2 **(E)** PTBP2 for PTC. **(F)** Immunohistochemistry (IHC) staining shown the expression of KHSRP, SRSF3, RBM9, NOVA2 and PTBP2 in normal thyroid tissues and PTC, data obtained from the HUMAN PROTEIN ATLAS database (HPA, https://www.proteinatlas.org/). **(G)** Comparison of the expression level of KHSRP, SRSF3, RBM9, NOVA2 and PTBP2 in normal thyroid tissues and PTC, data obtained from the Cancer Genome Atlas (TCGA, https://portal.gdc.cancer.gov) Student's t-test, ***p < 0.001; ns mean no significance. **(H)** Comparison of the expression level of KHSRP, SRSF3, RBM9, NOVA2 and PTBP2 in different stage (I, II, III and IV stage) of PTC from GEPAI database (http://gepia.cancer-pku.cn/).

**Figure 8:**
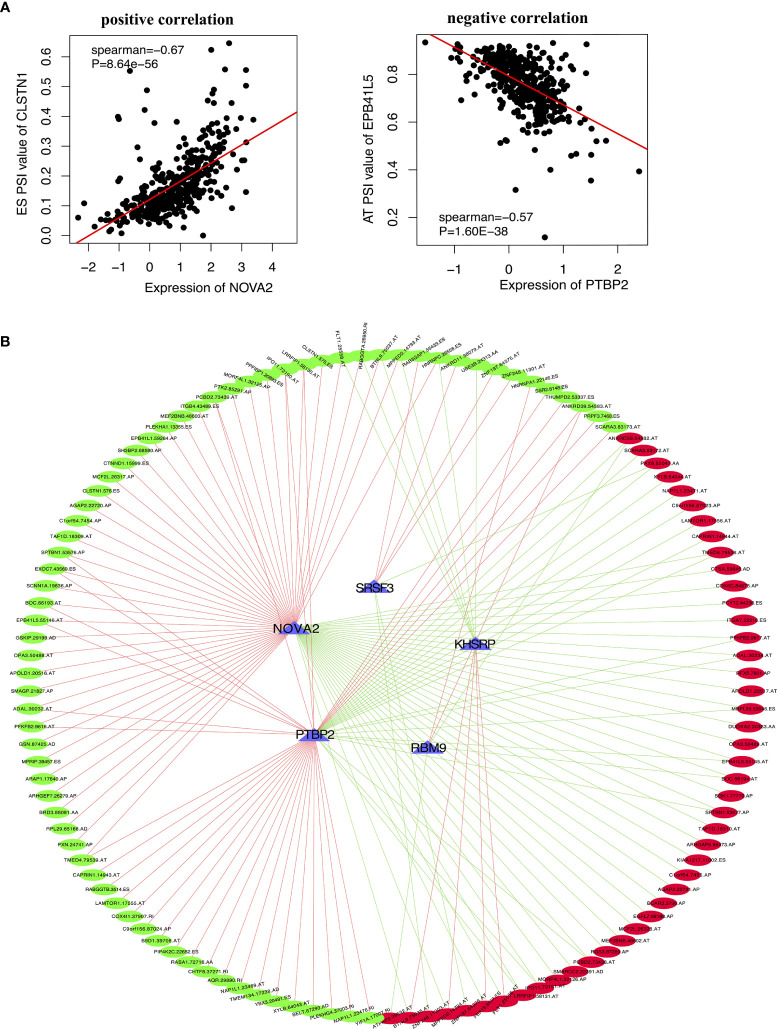
The correlation between the targeting SFs with AS events in PTC. **(A)** Two representative scatter plots showed the relevance between the expression of SFs and AS events, (left) positive correlation between NOVA2 and CLSTN1.575.ES PSI value, (right) negative correlation between PTBP2 and EPB41L5.55145.AT PSI value. **(B)** Interaction network of SF-AS (P<0.05, Spearman>0.4). Blue triangles represent RFS-associated SFs. Red lines represent SFs positively correlated with AS events, and green lines represent SFs negatively correlated with AS events. Red dots represent adverse AS events, and green dots represent favorable AS events.

In order to explore the upstream regulatory factors of dysregulated AS, the expression of 71 SFs was extracted from level 3 RNA-seq data of TCGA PTC. The results for univariate Cox regression analysis exhibited that 5 SFs (KHSRP, NOVA2, PTBP2, SRSF3 and RBM9) were significantly correlated to the RFS rate of PTC patients (**Supplementary Table 3**). The recurrence-free survival time curve with high and low expression of these 5 SFs shown as **Figures 7A**–**E**, among them, KHSRP was oncogenic factor, while NOVA2, PTBP2, SRSF3 and RBM9 were tumor inhibitor. We further searched The Protein Atlas database to detect the protein level of the 5 SFs in PTC. The immunohistochemistry (IHC) results showed that KHSRP, SRSF3 and RBM9 were located in nucleus, and the expression of SRSF3 and RBM9 were significantly lower in cancer than normal thyroid, while there was no significantly different between KHSRP, NOVA2 and PTBP2 in carcinoma and normal tissues (**Figure 7F**). The mRNA expression level of PTBP2 in normal tissues was higher than PTC, and NOVA2 expression was significantly involved in tumor stage, high expression in I-II stages and low expression in III-IV stage for PTC (**Figures 7G, H**). In addition, we used Spearman’s test to detect the relationship between the expression of these 5 SFs and PSI values ​​of RFS-associated AS events. The relationship network diagram showed that RFS-related 5 SFs (blue rectangles) were significantly associated with 117 AS events, with P value less than 0.05 and Spearman coefficient greater than or 0.4 as the cutoff value (p<0.05, Spearman≥0.4, **Figure 8B**). Interestingly, we found that the expression of KHSRP was positively correlated (red lines) with most of adverse survival prognostic AS events (red dots) but negatively correlated (green lines) with most of favorable AS events (green dots), however, the tumor suppressor SFs were inversely related to AS events. For example, **Figure 8A** exhibited the representative scatter plots of SFs and AS events correlation. Based on our preliminary exploration, we proposed the hypothesis that antineoplastic SFs play a key role in dysregulated AS, which may lead to tumor progression in PTC.

## Discussion

In this study, we first recognized diversified AS events with prognostic power using PSI of PTC AS data obtained from the TCGA. By using TCGA data, reported studies involved to PTC prognostic have shown that long noncoding RNA (lncRNAs), microRNA, and methylation data can work as prognostic factors. Chen et al., using binding motif data from Ensembl Biomart, predicted transcription factors (TFs) for affected genes to construct a TF/lncRNA/mRNA network, which predicted PTC prognosis with an AUC of 0.794 (28). Wang et al. established an N6-methyladenosine (m6A) RNA methylation-related risk signature of disease-free survival for a total PTC cohort with an AUC of 0.817. These models have also shown favorable prognostic predictions (29). We explored all events and established a ten-AS event signature to evaluate the RFS rate of PTC patients. The AUC values of the ROC curve for the 1-year, 3-year, 5-year and 10-year recurrence-free survival rate of PTC patients were 0.923, 0.916, 0.900 and 0.889 respectively, and we obtained more efficient prediction values from this model than with others. Importantly, the present ten-AS event signature has been confirmed with universality in predicting the prognosis of a wide range of tumors, including PRAD and LUAD patients.

AS is a key regulatory factor in the diversity of protein translation and gene phenotype, which is not only involved in normal physiological process but also plays an important role in the occurrence and development of human diseases, including PTC. For example, the alternatively spliced variant of thyroid stimulating hormoneβ (TSHβ), TSHβv (exon 2 deleted, exon 3 retained) has been associated with autoimmune thyroiditis in humans, which is also a high-risk factor for thyroid carcinoma (30). Circadian clock-independent AS events that play an important role in the homeostasis of the endocrine system, such as alternatively spliced *Clock* and* Bmal1*, are regulated by thyroid hormone receptor-associated protein 3 (THRAP3) and are closely associated with endocrine diseases including PTC (31, 32). Therefore, we could design different primers to evaluate the presence of AS events and types by PCR experiments and verified them by sanger sequence, which is relatively simple and effective.

In the ten-AS event prognostic prediction signature, some parental genes have been reported to play a key role in oncologic progression. Two spliced variants of *MARk3* (exon 16 included and exon 16 skipped) are differentially expressed by neural progenitors and neuronal cells and contribute to the important molecular regulation of cortical development (33). *TNFSF13*, a tumor necrosis factor, plays a significant role in tumor development and autoimmune diseases, and hypoxia promotes the retention of the intron of *TNFSF13* and suppresses the spliced isoform in MCF7 cells, which may contribute to a tumor suppressor effect (34, 35). *SEC14L1* with 3 alternatively spliced exons spanning exon 11 was specifically expressed in human peripheral blood leukocytes, and different protein isoforms may show differential expression in breast and ovarian cancer development (36). Nonetheless, few studies have reported the functional characteristic and of other parental genes in this prognostic signature. Moreover, we found that changes in the mRNA levels of the parental genes in most PTC samples were associated with patient prognosis. AS events can affect the level of transcription and proteome expression. Whether the change in mRNA levels is caused by the corresponding AS events needs to be verified with further experiments. However, there were no statistically significant associations between some genes and prognosis, and the loss or gain of regions resulting from AS events might produce meaningful biological behaviors. Therefore, the underlying molecular mechanisms of these AS events in the final model is unclear, and further functional experimental research is necessary.

In addition, we also explored the correlation between clinicopathological characteristics and the RFS rate of PTC patients, and the results of the univariate analysis demonstrated that age greater than 55 years, tall cell variant PTC, T3 and T4, lateral neck lymph node metastasis and pathological stage III and IV are indicative of poor prognosis. However, further multivariate analysis showed that the risk score of the ten-AS model was the only independent prognostic factor of PTC. In addition, we found that the subgroup of high-risk AS signatures was associated with age and tumor stage, which also showed that tumor-specific AS events play a role in cancer progression and metastasis. Moreover, biological function enrichment and pathway analysis of RFS-related AS events showed that cell-cell adhesion and the transforming growth factor beta receptor signaling pathway promote PTC tumor cell growth, invasion and metastasis (37, 38). KEGG enrichment revealed ribosome and transcriptional mis-regulation in cancer that was associated with the tumorigenesis and prognosis of PTC (39). Therefore, we hypothesized that carcinoma-related outcomes due to changes in AS may involve these pathways.

SFs are the main regulators of AS events and influence splicing sites by recognizing and binding precursor mRNAs. In our study, we identified five SFs related to the prognosis of PTC patients. *KHSRP* was reported to be oncogenic in non-small lung cancer, colorectal cancer and PTC (40–42). Overexpression of *KHSRP* activated IFN-αJAK-STAT1 signaling pathway and induced lung cancer cell invasion and metastasis (40). *KHSRP* might be a target mRNA regulated by the STAU1-mediated mRNA decay (SMD) pathway in PTC; however, the detailed mechanism is unclear (41). *NOVA2*, a key AS regulator of vascular morphogenesis, was overexpressed in lung carcinoma but its expression was negatively correlated with the prognosis of PTC patients in our analysis (43, 44). Finally, an obvious trend for SF-AS correlation network that the most of favorable prognostic AS events were positively correlated with the tumor suppressor SFs, while negatively correlated with oncogenic SFs expression, however, there was opposite relationship between adverse AS events and SFs. The role of 5 SFs in PTC and the regulation of alternative splice events remains to be verified by more experiments. This study provides a deeper understanding of the mechanism of SFs in the regulation and associated splicing patterns, which will help us to further explore the potential mechanism of AS events in the development and progression of PTC.

Although well performance of the present model has been watched, some limitations are inevitably existed in this study. First, this research lacks repeatability data that could be obtained from assessing the established prognostic predictors in other independent cohorts of PTC patients. Second, the prognostic significance of these potential therapeutic targets and diagnostic biomarkers for PTC still needs to be validated with further biological function experiments, mouse model and clinical trial. Nevertheless, our comprehensive analysis of recurrence-related SFs and AS events provides new knowledge and a new perspective for studying intrinsic molecular mechanisms and identifying potential therapeutic strategies for PTC.

## Conclusion

We performed a systematic analysis of AS events in PTC and constructed prognostic signatures that can be used to predict the recurrence-free survival rate for PTC patients. The ten-AS event signature involves genes including *SPHK2*, *SEC14L1*, *SLC22A17*, *CCL14*, *NUDT16*, *FXN*, *ADIRF*, *MARK3*, *TNFSF13* and *MTURN*, which can affect the prognosis and biological progression of PTC. The identification of prognosis-related AS events and SF regulatory network increases the understanding of the underlying mechanisms of PTC development and provides a new avenue for developing treatment strategies.

## Data Availability Statement

The original contributions presented in the study are included in the article/**Supplementary Material**. Further inquiries can be directed to the corresponding authors.

## Author Contributions

Conceptualization, ML, PH, SC, and RK. Resources, RK, ML, PC, HYH, NT, D-jO and BW. Data Curation, ML, RK, PC, HH, D-jO, Y-xZ, and NT. Writing, ML, D-jO and PH. Supervision, D-jO, PH, SC, and RK. Funding Acquisition, PH and SC. All authors contributed to the article and approved the submitted version.

## Funding

This work was supported by grants from the National Natural Science Foundation of China (81974423, 81902729), the Key Research and Development Programme of Hunan Province (2019SK2031), the Natural Science Foundation of Hunan Province of China (2020JJ5904) and China Postdoctoral Science Foundation (2020M672517).

## Conflict of Interest

The authors declare that the research was conducted in the absence of any commercial or financial relationships that could be construed as a potential conflict of interest.

The handling editor declared a shared affiliation with the authors at time of review.

## Publisher’s Note

All claims expressed in this article are solely those of the authors and do not necessarily represent those of their affiliated organizations, or those of the publisher, the editors and the reviewers. Any product that may be evaluated in this article, or claim that may be made by its manufacturer, is not guaranteed or endorsed by the publisher.
