# Supplementary material for: Comprehensive Analysis of Prognostic Alternative Splicing Signature Reveals Recurrence Predictor for Papillary Thyroid Cancer
Source: Front Oncol. 2021 Oct 13;11:705929. doi: 10.3389/fonc.2021.705929 (PMC8548661; doi:10.3389/fonc.2021.705929)
Supplement: Supplementary file 1 [file DataSheet_1.docx]

**Supplemental materials:**

**Comprehensive Analysis of Prognostic Alternative Splicing Signature Reveals Recurrence Predictor for Papillary Thyroid Cancer**

Mian Liu^1^, Rooh Afza Khushbu^1^, Pei Chen^1^, Hui-Yu Hu^1^, Neng Tang^1^, Deng-jie Ou-yang^1^, Bo Wei^1^, Ya-xin Zhao^1^, Peng Huang^1,2^* and Shi Chang^1,2,3^*

^1^Department of General Surgery, Xiangya Hospital Central South University, Changsha, China;

^2^Clinical Research Center For Thyroid Disease In Hunan Province, Changsha, Hunan, China;

^3^National Clinical Research Center for Geriatric Disorders, Xiangya Hospital Central South University, Changsha, Hunan, P.R. China

***Corresponding to** **Peng Huang**, Department of General Surgery, Xiangya Hospital Central South University, Changsha 410008, China. Tel: +86 15273124136, E-mail: xiangyahp@csu.edu.cn and **Shi Chang**, Department of General Surgery, Xiangya Hospital Central South University, Changsha 410008, China. Tel: +86 13973192319, E-mail: [changshi@csu.edu](mailto:changshi@csu.edu).


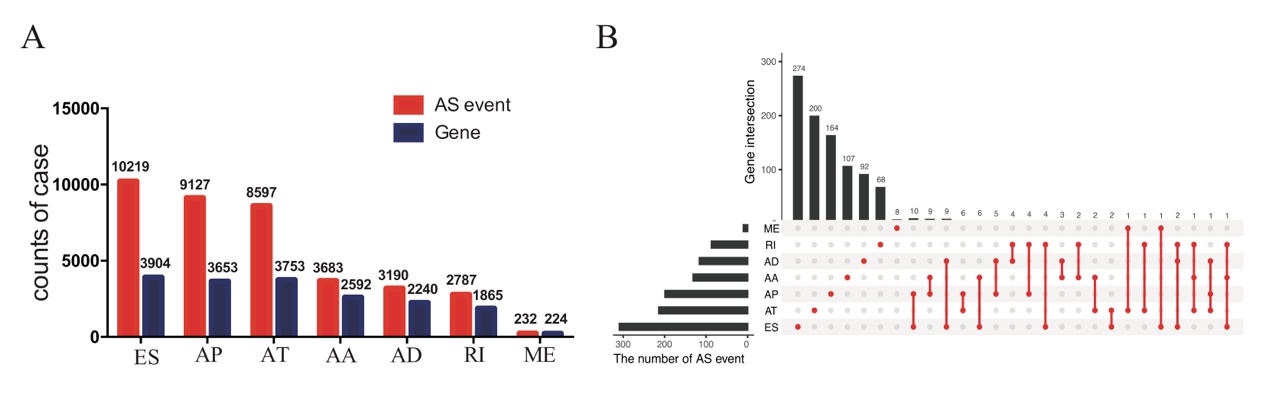


**Supplementary Figure 1**. The profile of 7 types of AS. **(A)** The number of AS events for each type and corresponding parental genes in PTC. **(B)** UpSet plots showed the interaction between 7 types of RFS-related AS events and parental genes in PTC.

**Supplementary Figure 2. LASSO coefficient profiles of RFS-associated AS events and ten-time cross-validation for tuning parameter selection in the LASSO model.**

**Supplementary Figure 3. Kaplan-Meier survival curves of SPHK2, SLC22A17, MARK3 and ADIRF.**


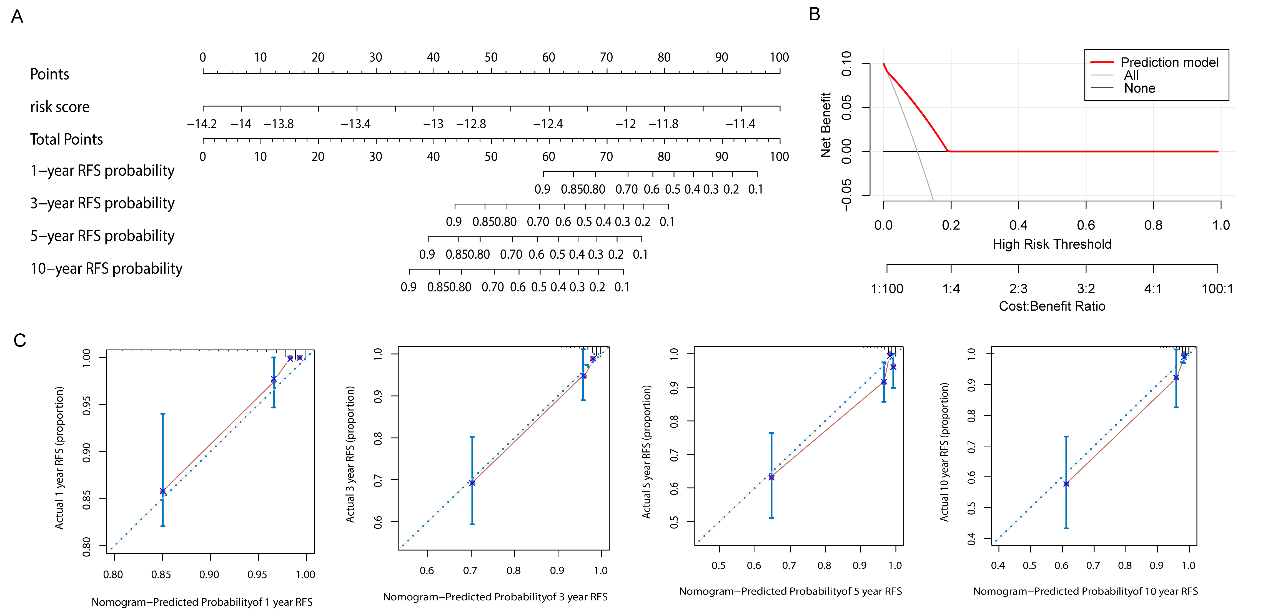


**Supplemental figure 4. Construction and validation of the nomogram model.** (A) Nomogram model for predicting the probability of 1-, 3-, and 5-year RFS in PTC patients. (B) Decision curve for nomogram to predict recurrence in PTC patients. (C) Calibration plots of the nomogram for predicting the probability of RFS at 1, 3, and 5 years.


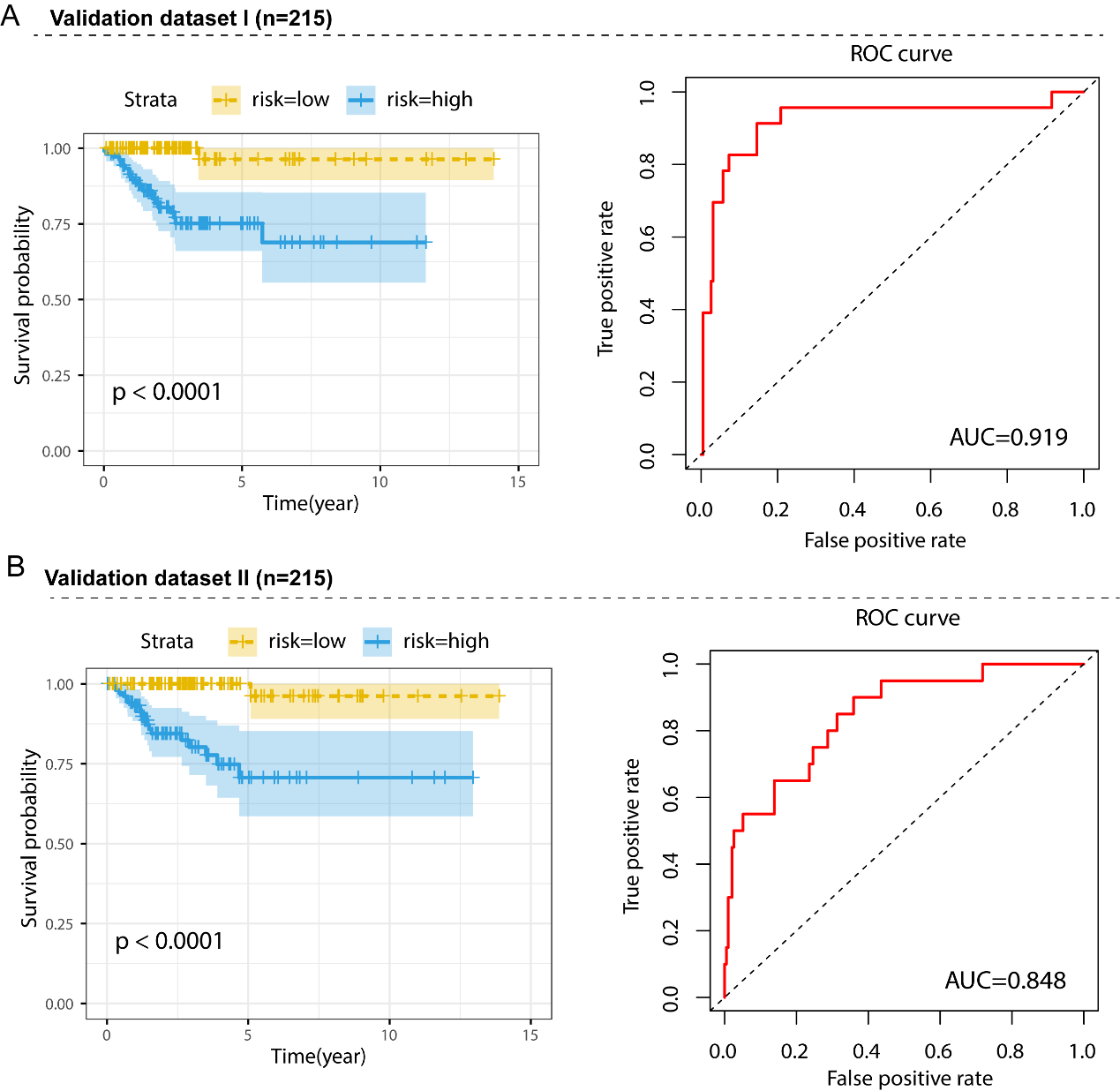


**Supplemental figure 5. Internal validation of ten-AS event signature.** To evaluate the performance of the model, the modeling dataset was random divided into two validation datasets (50 percent vs 50 percent, n=215), Kaplan-Meier survival curve and ROC curve were used to validate the performance of the ten-AS event signature in validation dataset I (A) and II (B).


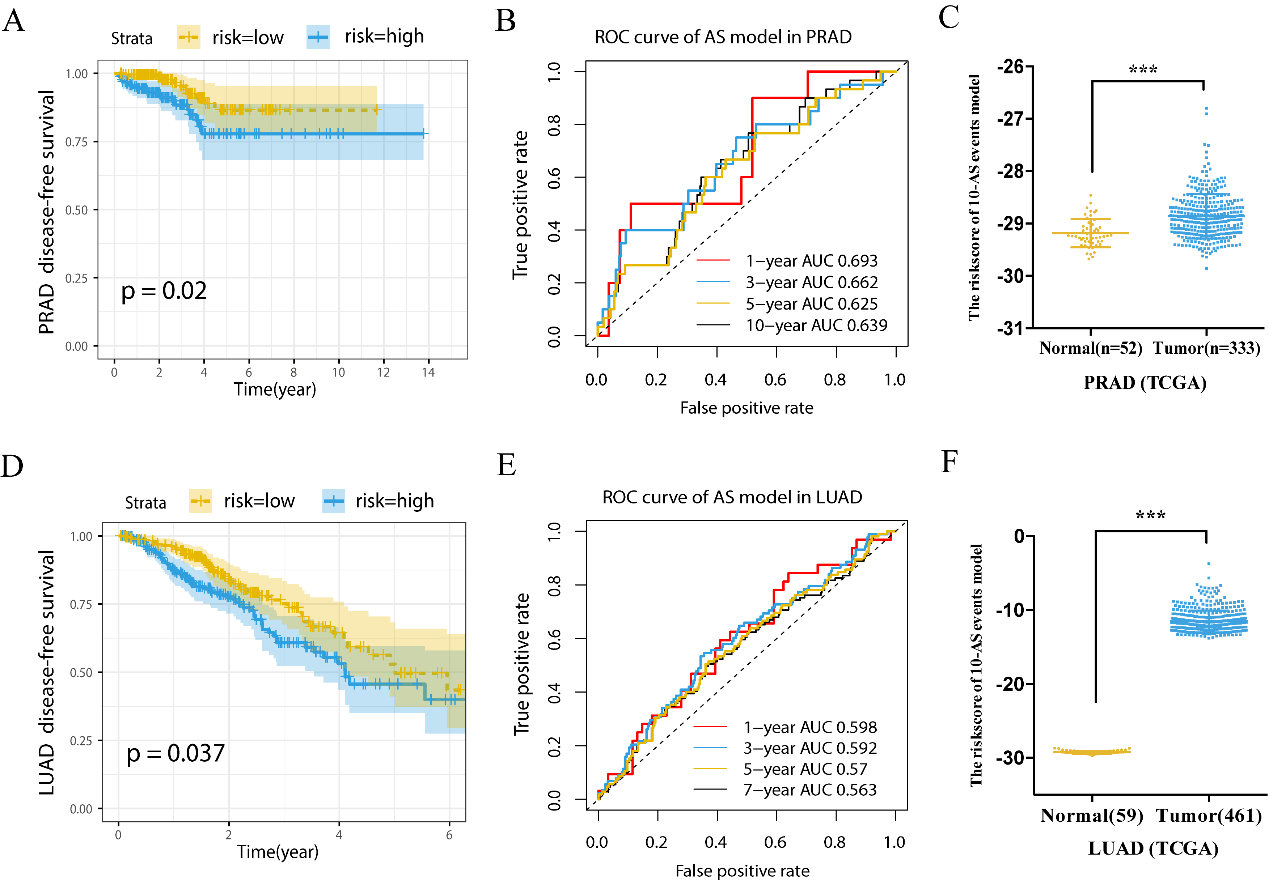


**Supplemental figure 6. The verification of prognostic value of the ten-AS event signature. (A)** Kaplan-Meier survival curve of the ten-AS prognostic predictor in prostate adenocarcinoma (PRAD). **(B)** ROC curve of the ten-AS prognostic predictor in PRAD. **(C)** The risk score of ten-AS prognostic predictor in normal prostate and carcinoma. **(D)** Kaplan-Meier survival curve of the ten-AS prognostic model in lung adenocarcinoma (LUAD). **(E)** ROC curve of the ten-AS prognostic predictor in LUAD. **(F)** The risk score of ten-AS prognostic predictor in normal and lung cancer.

Supplemental tables

S.table 1. Clinical Information of individals from cBioportal

S.table 2. Univariate analysis the association between each AS with RFS of PTC.

S.table 3. Univariate analysis the association between 5 SFs with RFS of PTC.

S.table 4. Comparison between cBioportal and GDC database.

S.table 5. Compare of AUC value of ROC curve among AS signatures
